# Usability and Overall Perception of a Health Bot for Nutrition-Related Questions for Patients Receiving Bariatric Care: Mixed Methods Study

**DOI:** 10.2196/47913

**Published:** 2023-11-08

**Authors:** Marina Beyeler, Corinne Légeret, Fabian Kiwitz, Klazine van der Horst

**Affiliations:** 1 Nutrition and Dietetics School of Health Professions Bern University of Applied Sciences Bern Switzerland; 2 Oviva AG Altendorf Switzerland; 3 University Children's Hospital Basel Switzerland; 4 Business Information Technology Zürich University of Applied Sciences Zürich Switzerland; 5 KIRATIK GmbH Sigmaringen Germany

**Keywords:** bariatric surgery, nutrition information, usability, satisfaction, artificial intelligence, health bot, mobile phone

## Abstract

**Background:**

Currently, over 4000 bariatric procedures are performed annually in Switzerland. To improve outcomes, patients need to have good knowledge regarding postoperative nutrition. To potentially provide them with knowledge between dietetic consultations, a health bot (HB) was created. The HB can answer bariatric nutrition questions in writing based on artificial intelligence.

**Objective:**

This study aims to evaluate the usability and perception of the HB among patients receiving bariatric care.

**Methods:**

Patients before or after bariatric surgery tested the HB. A mixed methods approach was used, which consisted of a questionnaire and qualitative interviews before and after testing the HB. The dimensions usability of, usefulness of, satisfaction with, and ease of use of the HB, among others, were measured. Data were analyzed using R Studio (R Studio Inc) and Excel (Microsoft Corp). The interviews were transcribed and a summary inductive content analysis was performed.

**Results:**

A total of 12 patients (female: n=8, 67%; male: n=4, 33%) were included. The results showed excellent usability with a mean usability score of 87 (SD 12.5; range 57.5-100) out of 100. Other dimensions of acceptability included usefulness (mean 5.28, SD 2.02 out of 7), satisfaction (mean 5.75, SD 1.68 out of 7), and learnability (mean 6.26, SD 1.5 out of 7). The concept of the HB and availability of reliable nutrition information were perceived as desirable (mean 5.5, SD 1.64 out of 7). Weaknesses were identified in the response accuracy, limited knowledge, and design of the HB.

**Conclusions:**

The HB’s ease of use and usability were evaluated to be positive; response accuracy, topic selection, and design should be optimized in a next step. The perceptions of nutrition professionals and the impact on patient care and the nutrition knowledge of participants need to be examined in further studies.

## Introduction

### Background

In terms of BMI, 42% of the Swiss population is overweight or obese [1]. To reach a sustainable weight reduction, restrictive and malabsorptive bariatric surgeries are one of the most effective methods [2,3]. Therefore, the number of procedures has more than quadrupled in the last 20 years [4]. To achieve a successful outcome of bariatric surgery, patients need to be provided with broad knowledge of food intolerances, dumping syndrome, and protein intake [5-10]. Therefore, patients need to be informed in detail before bariatric surgery to know what to expect and what kind of nutritional and behavioral changes must be made after the surgery [5]. To seek help for addressing these problems, patients use a variety of sources, such as websites [7,8]. These patients are in great need of satisfying and reliable answers to all their open questions [7,8,11,12]. In this regard, accessibility to and regular contact with a registered dietician are of utmost importance because they have been shown to remain the main source of reliable information, advice, and support for patients [7,8,11]. Preoperative dietetic counseling shows a positive effect on the outcome of bariatric surgery and benefits for weight loss [13,14]. In addition to preoperative counseling, the Swiss Society for the Study of Morbid Obesity and Metabolic Disorders highly recommends regular postoperative nutritional assessment and counseling [15]. Patients seem to need easy access to in-between dietetic consultations [7].

### Prior Work

Recent findings highlight the potential of novel artificial intelligence (AI)–based technologies, such as mobile phone apps and web-based platforms, in improving patient support and weight loss after bariatric surgery [7,16,17]. Versteegden et al [17] showed that eHealth platforms used postoperatively, with topics such as information dissemination regarding obesity and bariatric surgery, can lead to significantly greater weight loss at 1 and 2 years postoperatively. In addition, there is a specific recommendation for combining accessible information for patients with obesity in electronic and nonelectronic media [18]. A recent study [19] showed good acceptance and usability of a smartphone app for postoperative care for bariatric surgery. This program was based on a standardized questionnaire, which patients completed in the app periodically, as well as reminders and push notifications to take supplements and engage in physical activity. In general, web-based health information is a support for patients and can potentially lead to more productive conversations with health care professionals, as frequently asked questions (FAQs) can already be answered before a visit [20]. Furthermore, it is an opportunity to provide evidence-based support for patients who do not require an expensive and time-consuming visit with a health care professional but nevertheless need information and advice in between visits with the responsible dietician [21,22].

People have access to information in all areas around the clock. Incorrect information about nutrition can easily be found in chat rooms on social media, and the amount of information on the internet can be overwhelming for patients [6,20,23,24]. Evidence-based health bots (HBs) could potentially fill the gap in providing assistance and information while preventing patients from consuming incorrect information on other internet-based platforms [12,24]. Current evidence on health chatbots and AI shows that they mainly focus on nutritional and neurological disorders [25], physical activity [26,27], and mental health [27]. Future research studies should address the concern of the lack of data about the acceptability and usability of patient-centered eHealth tools among patients [12,20,23,28-30]. Although the possible benefits of an HB are seen in creating more time for dieticians to focus on behavioral and individual support, as simple knowledge questions can be cleared by the HB [12,17,21,22,24], no studies are available on the use of an HB in the dietetic treatment of patients in the bariatric setting. Therefore, this study aimed to explore how patients receiving bariatric care rate the overall usability, benefits, risks, strengths, and weaknesses of and trust in an HB for nutrition-related questions before and after a bariatric intervention. The second aim was to evaluate how patients receiving bariatric care rated the quality of the answers generated by the HB to their nutrition-related questions [31].

## Methods

### Development of the Knowledge Corpus and HB

**Figure 1:**
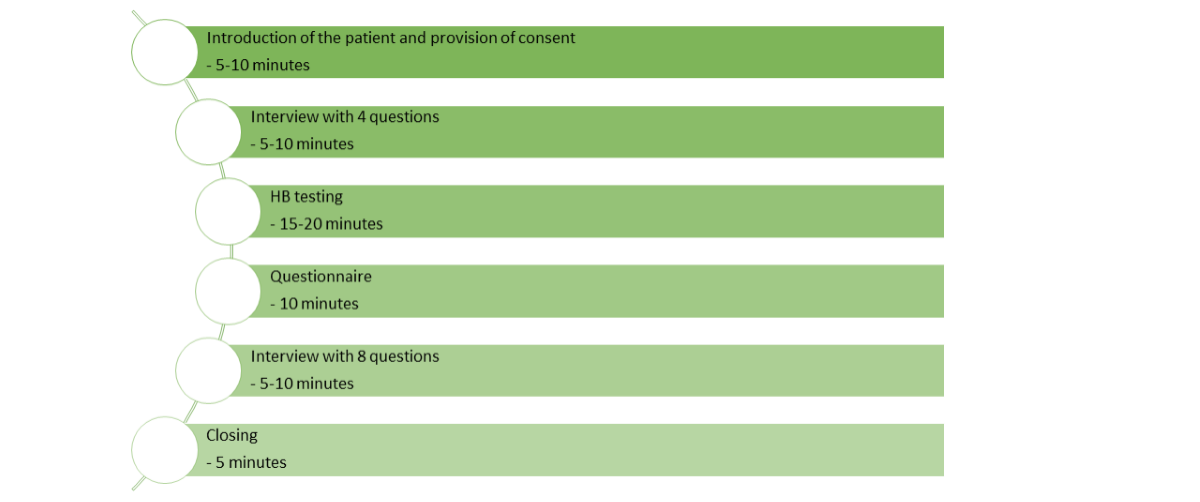
Study procedure. HB: health bot.

The elaborated knowledge corpus was developed based on patient documents from the collaborating clinic and FAQ sheets from various bariatric centers in Switzerland. Two feedback loops, the incorporation of the collected feedback, and 3 fine-tuning iterations were carried out during the development of the HB. The feedback loops were conducted with the help of experienced nutritionists. The technology used was Hugging Face [32], which is an AI specialized in recognizing same sentences. This model was designed to compute sentence embeddings for English and German texts. The question that is entered in the HB by the user is compared with the questions in the model. The question that is most similar to it is used, and its answer is communicated to the user. This means that AI does not learn the questions but just simply hand overs questions and answers. The latest HB version was tested in a patient study (Beyeler, M. unpublished data, 2022) with 161 questions and showed the following outcomes: 85 (52.8%) questions were answered correctly by the HB, and 76 (47.2%) questions were not answered satisfactorily. Of these 76 questions, 36 (47%; 22.4% of the total questions) were not included in the knowledge corpus and, therefore, could not be answered, and 40 (53%; 24.8% of the total questions) questions were included in the knowledge corpus but provided with a nonmatching answer. In Figure 1, an example of an HB-generated answer is presented.

### Study Design

A study evaluating the usability and performance of an HB was conducted, in which quantitative and qualitative methods are applied independently [33-35]. The study was conducted via face-to-face interviews with patients with obesity in the preoperative and postoperative settings, which took approximately 45 minutes. The study took place in a bariatric center. First, a short qualitative interview with 4 questions was conducted. The second task was the testing of the HB, wherein the participants asked the HB nutrition-related questions. Regarding the following predefined categories, which correspond to the structure of the HB’s knowledge, at least 1 question per category should have been asked per person: postoperative diet plan, mealtime rhythm, protein, dumping syndrome, liquids, food tolerance, vitamins, digestion, quantity of food, and others. In “others,” the participants were free to ask any other bariatric nutrition–related questions. Participants were also encouraged to ask more than 1 question per category to be able to generate a higher quantity of questions, which could possibly be included in a further development cycle. The questions’ content and wording were generated by the participants. The satisfaction with the answers of the HB had to be evaluated after each question. After the testing phase, participants completed a web-based questionnaire with 46 items. At the end, another qualitative interview with 8 questions was conducted. The study procedure is illustrated in Multimedia Appendix 1.

### Sample

In all, 12 participants were recruited from September 2021 to January 2022 at a specialized bariatric center in Berne, Switzerland. Potential patients who entered the bariatric center had their first appointment with a specialized medical physician, followed by various medical clarifications, including nutritional counseling from a dietician. In this counseling session, the patients were asked whether they were interested in participating in the study. In case of consent, the potential study participants were contacted by the research team for an appointment and to clarify their questions.

Eligible participants were defined as adults (aged ≥18 y) with obesity (BMI≥35 kg/m^2^) from Switzerland who were planning to undergo a Roux-Y gastric bypass or sleeve gastrectomy bariatric surgery in the next 3 months or who had undergone one of the mentioned surgeries within the last 2 months. As comparable usability studies with 7 to 21 participants achieved a high detection rate, we decided to select a sample of 10 to 14 participants with an equally distribution of patients before surgery and patients after surgery [31,33,36-39]. Participants were selected based on the need for bariatric surgery (Roux-Y gastric bypass or sleeve gastrectomy) according to the Swiss Society for the Study of Morbid Obesity and Metabolic Disorders criteria [40]. In addition, potential participants had to be proficient in German, as the HB was available only in the German language. Furthermore, patients must have had at least 1 preoperative dietetic counseling session. This ensures basic knowledge about bariatric nutrition among participants, which is helpful for getting ideas about what questions to ask the HB [5,10,11]. For participants after surgery, the time frame for the survey was up to 2 months after surgery, as the HB’s knowledge base was primarily developed for this period because most adaptations to the patient’s diet must be made within the first 2 months after surgery [10,11]. Patients with obesity who received conservative or drug-related weight reduction therapy were excluded.

### Ethical Considerations

As a usability study bears only very minimal risks for the participants, no ethics approval was required [41], as confirmed by the Business Administration System for Ethics Committees, which rejected jurisdiction (Business Administration System for Ethics Committees–Nr: Req-2021-00952). Therefore, this study was not registered at ClinicalTrials.gov.

All individuals participated voluntarily and did not receive monetary compensation. They were free to withdraw their participation at any time. An informed consent form, which included information about the study aim and methodology, was signed by the participants before participation. Other than the inclusion criteria, there was no collection of health-related data in this study.

### Qualitative Interviews and Analysis

The study consisted of a qualitative part, which was conducted by MB using 4 questions at the beginning of each session with the participant and 8 questions at the end of the session. These items were specifically developed for this study and are presented in Textbox 1. The interviews aimed to gain deeper insight into participants’ perceptions of the HB. In addition, the topics “perception,” “strengths,” “weaknesses,” and “further development” were explored, which could be better embedded in an interview than in a questionnaire. After the first patient interview, small adaptation to the interview questions were made for improvement. The 2 interview sequences were recorded with a smartphone and named under the participant’s assigned ID as part 1 or 2. The audio recordings were then saved locally on a laptop for further processing and deleted from the smartphone. With the support of the f4transkript (audiotranskription) software, MB created semantic content transcripts from the interviews according to the simple transcription rules of Kuckartz [42]. Subsequently, a summary inductive content analysis according to Mayring [43] was performed. This step was performed manually, and the data were entered into an Excel (Microsoft Corp) database. The focus of this further processing was on summarizing and paraphrasing the transcripts, with the goal of concentrating the content and formulating summarized answers by topics.

**Qualitative interview questions asked before and after the testing of the health bot (HB).**

**Before testing**

- Try to imagine an HB to answer nutrition-related questions in bariatrics. What would be important to you about it?
- What topics or questions would it need to help you with?
- What benefits would you hope to gain from an HB?
- What should not happen when using an HB?

**After testing**

- What was it like for you in general to use the HB?
- What do you think are the strengths of the HB?
- What do you think are the weaknesses of the HB?
- Do you have any concerns about using the HB?
- What content adjustments or enhancements would you make?
- What general adjustments would you make?
- What would be needed for further development?
- Could the feedback be made complete? If not, what would you like to add?

### Quantitative Data Collection

The questionnaire for quantitative data collection was built in a web-based survey tool called UmfrageOnline, which is available only through encrypted connections [44]. The questionnaire was divided into 2 segments. The first segment consisted of the System Usability Scale (SUS) [45] validated in German, which is the main spoken language at the location of the survey’s execution [46]. The SUS consists of 10 items and is one of the most widely used standardized usability questionnaires [47,48]. The answers are ranked on a 5-point Likert scale [49], with positive and negative formulations alternating to prevent response bias [47,50]. Because the SUS did not cover all topics of interest for this study, a second part of the questionnaire was created. A total of 4 frequently used usability and acceptability questionnaires—the Telehealth Usability Questionnaire [51]; Service User Technology Acceptability Questionnaire [52,53]; Usefulness, Satisfaction, and Ease of Use Questionnaire [54]; and Post-Study System Usability Questionnaire [55]—were selected and evaluated according to the research question as well as the HB functionalities. After removing redundant and duplicate items, 28 of the total 92 items were selected and used in the questionnaire. According to the usability study by Li et al [56], 2 items each from the categories “intention to share information” and “intention to seek information” were also added [56]. According to the categories used in the abovementioned questionnaires, the final items were assigned to the following dimensions: usability (3 items), usefulness (6 items), user-friendliness and learnability (6 items), interface quality (4 items), reliability (1 item), satisfaction (4 items), risks (2 items), benefits (2 items), intention to share (2 items), and intention to seek (2 items). Similar to most of the used sources, the answer options of the questionnaire were presented on a 7-point Likert scale [51-57]. Furthermore, 4 demographic questions, namely those on sex, age, highest level of education, and digital ability, were included at the end of the questionnaire [36-38,58].

### Ratings of the Answers of the HB

To obtain quantitative data about the participant’s satisfaction with the answers the HB provided in the testing, participants were asked to rank each answer. A 5-point Likert scale was included right below the answer, with the following options: very good (1), good (2), acceptable (3), bad (4), and very bad (5). Participants were asked to rate the answers according to their personal satisfaction.

### Data Analysis

Data processing and statistical analysis were performed in R Studio (version 3.6.1; R Studio Inc), with attached base packages *GlobalEnv*, *tools:rstudio*, *package:stats*, *package:graphics*, *package:grDevices*, *package:utils*, *package:datasets*, *package:methods*, *Autoloads*, and *package:base* [59]. To determine the SUS score, which ranges from 0 to 100, each answer option was assigned a number from 0 to 4, taking the positive or negative formulation of the question into account. All items were summed up, and the resultant was multiplied by 2.5 [47]. The interpretation of the SUS score was based on the study by Bangor et al [60], with the highest score being 100 [60]. For the SUS, 1 patient was excluded from the evaluation because they got confused with the questions phrased alternately positive and negative. For the remaining part of the questionnaire, the participant ensured that the questions were read carefully and was able to answer correctly.

The second part of the questionnaire was analyzed through descriptive statistics of each item, namely mean and SD. The response options ranged from 1 to 7, with 1 representing “strongly disagree” and 7 representing “strongly agree.” Each dimension in the questionnaire (eg, benefits) was presented separately, with mean and SD calculated for each dimension [61]. The internal consistency of the dimensions with at least 3 items was analyzed using Cronbach α [62]. The dimensions “usability” (Cronbach α=.87), “usefulness” (Cronbach α=.92), “user-friendliness and learnability” (Cronbach α=.91), and “satisfaction” (Cronbach α=.95) showed very good values (raw Cronbach α>.8), and “interface quality” showed an acceptable value (Cronbach α=.61). To explore a possible correlation between digital affinity and the different categories, Spearman correlations were calculated [63]. Owing to the small cohort size, the mean values of all categories were compared between the before surgery and after surgery groups using the nonparametric Mann-Whitney *U* test [63].

The number and percentage of questions asked in the HB that fell under each category, as well as for the received score from 1 (very good) to 5 (very bad) were calculated.

## Results

### Patient Characteristics

Table 1 presents an overview of the characteristics of the 12 participants included in this study. Among the 12 patients, 8 (67%) were female, and the majority (n=10, 83%) were aged between 18 and 49 years. Education was evenly distributed. For self-assessed digital affinity, which was scored 1 (none) to 10 (expert), the mean score was 6.9 (SD 1.98). Of the 12 participants, 7 (58%) were in the preoperative phase, and 5 (42%) were in the postoperative phase.

**Table 1:** Characteristics of the study participants (N=12).

Characteristic			Values
Sex (female), n (%)			8 (67)
**Age group (y), n (%)**			
	18-29	3 (25)	
	30-39	5 (42)	
	40-49	2 (17)	
	50-59	1 (8)	
	60-69	1 (8)	
**Highest level of education, n (%)**			
	Compulsory elementary school	1 (8)	
	Vocational apprenticeship	3 (25)	
	Higher technical or vocational education	4 (33)	
	Bachelor’s or master’s degree or degree in business administration	3 (25)	
	Apprenticeship, vocational baccalaureate, or professional certificate	1 (8)	
**Phase of operation, n (%)**			
	Before operation	7 (58)	
	After operation	5 (42)	
Digital affinity (0-10), mean (SD; range)			6.9 (1.98; 2-10)

### Quantitative Results

The Mann-Whitney *U* test (Table 2) showed no significant difference between the before surgery and after surgery groups in scores for any item, including the SUS (*P*=.06; the *P* values ranged from .13 to >.99), so these groups were combined as 1 sample group for the analyses. The median SUS score in the study was 90 out of 100, and the mean SUS score was 87 (SD 12.5). Both values are classified as “excellent” [60]. The range of the 11 individual scores was from 57.5 to 100. The other dimensions are listed in Table 3. The dimension “usability” showed the highest mean value, with 6.47 (SD 1.16) out of 7 points on the Likert scale. The highest per item mean value of 6.75 (SD 0.87) was reached by the item “The HB is simple and easy to understand” (“interface quality”). In the same dimension, the item “The HB can do everything I want it to do” scored the lowest, with a mean of 4.75 (SD 1.76; “interface quality”). All dimensions showed high means, ranging from 6.47 (SD 1.16) for usability to 5.28 (SD 2.02) for usefulness, showing the positive perceptions of the participants. The dimension “risk” was worded negatively, so the score 1 is the highest possible score, and 7 is the lowest possible score; it showed a low risk with a mean of 1.58 (SD 1.56). The items “The HB meets my needs” and “The HB can do everything I would want it to be able to do” scored the lowest, with mean values of 4.75 (SD 2.18 and SD 1.76, respectively). No significant correlations were observed between digital affinity and the measured acceptability dimensions with *P* values ranging between .41 and .86 (Multimedia Appendix 2).

**Table 2:** Results of the Mann-Whitney U test for the comparison of the usability and perception dimensions between patients before bariatric surgery and patients after bariatric surgery.

Category	Mann-Whitney *U* test	*P* value^a^
SUS^b^ (usability)	19	.39
Usability	17	>.99
Usefulness	17	>.99
User-friendliness and learnability	17	>.99
Interface quality	18.5	.93
Reliability	5	>.99
Satisfaction	20.5	.68
Risks	10	.20
Benefits	16.5	.93
Intention to share information	27.5	.11
Intention to seek information	24	.27

^a^The exact significance was used because of the small sample size.

^b^SUS: System Usability Scale.

**Table 3:** Means and SDs of the questionnaire items.

Category and item			Values, mean (SD)^a^
**SUS^b^ (overall score)**			4.48 (0.73)
	I think that I would like to use the Bariatric Nutrition Health Bot frequently.	4.09 (1.08)	
	I found the Bariatric Nutrition Health Bot unnecessarily complex.	4.82 (0.39)	
	I thought the Bariatric Nutrition Health Bot was easy to use.	4.18 (1.27)	
	I think that I would need the support of a technical person to be able to use the Bariatric Nutrition Health Bot.	4.91 (0.29)	
	I found the various functions in the Bariatric Nutrition Health Bot were well integrated.	4.09 (1.24)	
	I thought there was too much inconsistency in the Bariatric Nutrition Health Bot.	3.91 (1.08)	
	I would imagine that most people would learn to use the Bariatric Nutrition Health Bot very quickly.	4.45 (0.89)	
	I found the Bariatric Nutrition Health Bot very cumbersome (awkward) to use.	4.82 (0.39)	
	I felt very confident using the Bariatric Nutrition Health Bot.	4.55 (0.66)	
	I needed to learn a lot of things before I could get going with the Bariatric Nutrition Health Bot.	5.00 (0.00)	
**Usability (overall score)^c^**			6.47 (1.16)
	I was able to perform the tasks quickly using the HB^d^.	6.5 (1.45)	
	I was able to perform the tasks efficiently using the HB.	6.33 (1.23)	
	I felt comfortable using the HB.	6.58 (0.79)	
**User-friendliness and learnability (overall score)^c^**			6.26 (1.5)
	It was simple to use the HB.	6.58 (1.44)	
	It was easy to learn to use the HB.	6.67 (1.15)	
	I believe I could become productive quickly using the HB.	6.25 (1.22)	
	The HB is user-friendly.	5.92 (1.93)	
	Using the HB is effortless.	6.58 (0.9)	
	Both occasional and regular users would like to use the HB.	5.58 (2.02)	
**Interface quality (overall score)^c^**			5.69 (1.78)
	The way I interact with the HB is pleasant.	5.67 (1.78)	
	I like using the HB.	5.58 (2.07)	
	The HB is simple and easy to understand.	6.75 (0.87)	
	The HB can do everything I would want it to be able to do.	4.75 (1.76)	
**Reliability**			
	Whenever I made a mistake using the HB, I could recover easily and quickly^e^	5.5 (1.64)	
**Usefulness (overall score)^c^**			5.28 (2.02)
	The HB improves my access to nutrition services.	5.42 (1.78)	
	The HB saves me time traveling to a hospital or specialist clinic.	5 (2.52)	
	The HB covers my nutritional counseling needs.	4.92 (1.78)	
	The HB is useful.	5.75 (1.82)	
	The HB saves me time when I use it.	5.83 (2.12)	
	The HB meets my needs.	4.75 (2.18)	
**Satisfaction (overall score)^c^**			5.75 (1.68)
	The HB is an acceptable way to receive nutrition information.	6 (1.41)	
	I would use the HB again.	5.83 (1.99)	
	Overall, I am satisfied with the HB.	5.5 (1.83)	
	The HB can be trusted to work appropriately.	5.67 (1.61)	
**Risks (overall score)**			1.58 (1.56)
	The HB has made me feel uncomfortable (physically or emotionally)^f^	1 (0)	
	The HB makes me worried about the confidentiality of the private information being exchanged through it.	2.17 (2.08)	
**Benefits (overall score)**			6.13 (1.3)
	The HB can be/should be recommended to people in a similar situation as I am.	5.83 (1.47)	
	The HB is certainly a good addition to my regular nutrition counseling care.	6.42 (1.08)	
**Intention to share information (overall score)**			5.92 (1.25)
	I am willing to share nutrition related information with the HB.	6.08 (1.31)	
	I am willing to share nutrition related information with the HB in the future.	5.75 (1.22)	
**Intention to seek information (overall score)**			5.96 (1.65)
	I am willing to seek nutrition related information via HB.	6 (1.54)	
	I am willing to seek nutrition related information via HB in the future.	5.92 (1.83)	

^a^Possible scores range from 1 to 5, and negative or positive items are aligned.

^b^SUS: System Usability Scale.

^c^Possible scores range from 1 to 7.

^d^HB: health bot.

^e^6 missing values: no answer could be given because troubleshooting was not necessary.

^f^No SD because all values were at 1, and correlation calculation was not possible.

### Ratings of the Answers of the HB

Patients asked most questions in the liquids category, followed by the dumping syndrome category. The possible ratings for the HB’s answers ranged from 1 (very good) to 5 (very bad). If the topic “other” was excluded, the average score of all ratings was 2.3 (SD 0.4). The “dumping syndrome” category had the most ratings of 1 (“very good”; 13/19, 68%). If the ratings 1 (very good) and 2 (good) are combined, the “protein” category received the best ratings, with 83% (10/12) of the rated answers receiving a 1 or 2. The answers of the HB on questions about “food tolerance” were rated as having the lowest quality, with the most ratings of 5 (3/11, 27%) and the most ratings of 4 (bad) and 5 (very bad) combined (5/11, 45%). In the “others” category, patients asked questions about preoperative nutrition, the allowance of specific food groups and ingredients, and blood glucose and sugar intake. An overview of the ratings is displayed in Table 4.

**Table 4:** Ratings of the generated answers of the health bot^a^.

Topic	Questions asked (n=162), n (%)	Score, mean^b^ (SD)	Score 1, n (%)	Score 2, n (%)	Score 3, n (%)	Score 4, n (%)	Score 5, n (%)
Postoperative diet plan	15 (9.3)	1.9 (1.2)	8 (53.3)	3 (20)	1 (6.7)	3 (20)	0 (0)
Mealtime rhythm	12 (7.4)	2.1 (1.2)	5 (41.7)	3 (25)	3 (25)	0 (0)	1 (8.3)
Protein	12 (7.4)	1.9 (1.1)	5 (41.7)	5 (41.7)	1 (8.3)	0 (0)	1 (8.3)
Dumping syndrome	19 (11.7)	1.8 (1.3)	13 (68.4)	1 (5.3)	1 (5.3)	3 (15.8)	1 (5.3)
Liquids	25 (15.4)	1.9 (1.2)	13 (52)	5 (20)	4 (16)	2 (8)	1 (4)
Food tolerance	11 (6.8)	3.2 (1.5)	2 (18.2)	2 (18.2)	2 (18.2)	2 (18.2)	3 (27.3)
Vitamins	13 (8.0)	2.5 (1.3)	4 (30.8)	3 (23.1)	3 (23.1)	2 (15.4)	1 (7.7)
Digestion	14 (8.6)	2.6 (1.3)	4 (28.6)	2 (14.3)	5 (35.7)	1 (7.1)	2 (14.3)
Quantity of food	16 (9.9)	2.6 (1.3)	4 (25)	4 (25)	4 (25)	3 (18.8)	1 (6.3)
Inter total	137 (84.6)	2.3 (0.4)	58 (42.3)	28 (20.4)	24 (17.5)	16 (11.7)	11 (8)
Others	25 (15.4)	3 (1.1)	3 (12)	4 (16%)	9 (36)	7 (28)	2 (8)
Total	162 (100)	2.4 (0.5)	61 (37.7)	32 (19.8)	33 (20.4)	23 (14.2)	13 (8)

^a^N values for the scores can be found in the second column (ie, questions asked).

^b^Possible scores are as follows: 1 (very good), 2 (good), 3 (acceptable), 4 (bad), and 5 (very bad).

### Qualitative Results

#### Important Aspects and Benefits of an HB

Patients mentioned that an HB should be relevant to everyday life, give examples for the implementation of the diet, and be able to provide specific information about certain food products. In addition, an HB should provide answers that are easy to understand, detailed, and correct in content. Furthermore, the ease of use should be a given:

That the answers are simple, understandable, but also that my questions are answered well, that it has a relation to the question that I ask. And above all, that it is easy to understand.

ID 05

Coverage of a variety of topics, including topics beyond nutrition, was a need for patients. Explicitly desired topics included the following: diet structure, food choices, specific product information, dumping syndrome, blood glucose, types of sugar, eating and drinking amounts, protein, food aversions, complications, preoperative nutrition, and mealtime rhythm:

...so just roughly information, before and then especially the diet after the surgery...Specifically with food, what is good, what is not good.

ID 07

Some benefits of using an HB were observed. Support in everyday life, time saving, lower inhibition threshold for receiving information, constant availability, autonomy, relief of in-person nutrition counseling burden, and reliable sources of information were mentioned:

The advantage is certainly, if you have such a tool, you know where to go to look something up...a program, where you can go on and know, there are real things in it, the facts...

ID 11

Simply it’s about efficiency. You already have like a first point of contact before you call the doctor or something. It is faster. I think it also relieves the doctor if a few questions can be clarified beforehand.

ID 01

#### Strengths and Weaknesses of the HB

The HB was viewed as a good tool for supporting patients with obesity. The strengths of the HB were perceived in its user-friendliness, anonymity, practicability, accessibility, free formulation of questions, provision of a variety of topics, and correct or detailed answers:

So it’s very user-friendly, very simple...I think it’s a great idea...Yes you can see that it is not yet fully developed, but actually so the basic idea and the user-friendliness I find very good.

ID 03

The strength of the Bot is that you can certainly type in the question the way you actually just want to say it and it finds an answer to it relatively well.

ID 11

By contrast, the design, the presentation of the answers, presence of some technical terms in the answers, and the lack of knowledge of the HB were mentioned as weaknesses. Some answers did not fit well with the questions or were too unspecific, or examples within the answers were missing:

...I’m also someone who looks at the visual part as well and it was almost too simplistic for me, compared to other apps.

ID 02

The answers were not always satisfactory. I asked a question once and then a completely different answer came. And then when I asked another question, the answer just came to the first question. That’s not quite right yet.

ID 03

#### Potential Development Needs for the HB

For the further development of the HB, the following topics were mentioned, which should be considered: specific product information, meal or snack composition, allowance of certain foods or food groups, blood sugar, sugar types, long-term nutrition, preoperative nutrition, more examples or meal ideas, including different types of diets (eg, vegetarian and vegan), and integrating an FAQ as an addition. Further, the HB should provide more detailed answers on some existing topics (digestion postoperatively, vitamins, and meal spacing). For some participants, expanding the content outside nutrition was desirable:

...maybe, I don’t know if you could individually cater to certain diets, so someone who eats vegetarian or vegan or only without fish or whatever or has any food intolerances. This is certainly also special after surgery, where you pay a little bit more attention.

ID 12

The following general adjustments to the HB were mentioned: optimize the response accuracy; add visuals; add a glossary of technical terms; improve the design, structure, and readability; add a history of asked questions; provide print function; make the HB mobile app based, add a topic breakdown, slightly optimize usability, provide the possibility to look up what was asked before:

...that if this question comes up again, then I see that I have already asked it, I actually already know that.

ID 05

## Discussion

### Principal Findings

#### Overview

This study showed that the usability of the HB was overall rated as excellent in the SUS and that the other dimensions were rated positive, such as usefulness and “interface quality.” In line with these results, the qualitative data revealed the patients’ perception of the HB as having desirable usability, simple operation, and easy comprehensibility. The overall usability was found to be good. The 2 lowest rated items in the mentioned categories were “The HB meets my needs” and “The HB can do everything I would want it to be able to do.” This was expected, as the HB in this study is still in an early developing stage. Another interpretation of the 2 lowest rated items may be that the HB cannot replace a consultation with a dietitian for patients, as was cited as a concern by one of the participants in the interview. However, several people mentioned assistance in everyday life and lower inhibitions to access the HB rather than calling the health care practice as advantages. The HB was applied to the time between consultations, during which patients have the need to receive helpful information [7], instead of replacing a consultation. This coincides with the idea behind the HB, which was for the HB to be an addition to the already well-standardized and proven face-to-face consultations by a dietician. A combination of face-to-face appointments and digital access to information between the appointments might be a good solution for providing better support to patients with obesity [64]. The questionnaire showed highly esteemed benefits from the HB, as the items “The HB can/should be recommended to people who are in a similar situation as I am” and “The HB is certainly a good addition to my regular nutrition counseling care” achieved high scores.

Furthermore, the mentioned advantages of an HB were the ease of obtaining reliable information on the web, opportunity to save time, constant availability, more autonomy as a patient, and thus relief of the burden on dieticians. Similar points were confirmed from the perspective of dietitians in the study by Elvin-Walsh et al [7], whereas Nadarzynski et al [12] confirmed similar aspects in an HB acceptance study. In this study of Nadarzynksi et al [12], the users had a positive view of the anonymity of the HB [12], which goes hand in hand with the lower threshold to disclose more intimate or uncomfortable aspects of health to the HB than to a dietician in face-to-face counseling. Most patients with obesity also seem to prefer having access to information via smartphones, which underlines the constant availability of and access to information [65-67].

#### Perceived Trust in and Strengths and Weaknesses of the HB

All participants negated the item “The HB makes me feel uncomfortable (physically or mentally).” The confidentiality of privacy (“I am concerned about the confidentiality of private information shared through the HB”) was rated slightly positive, which is relatable to the concerns mentioned in the interviews. Several participants addressed the privacy and confidentiality of the entered questions. This was also found in previous studies, where people were unsure about using a chatbot as part of their health care because of the questioned quality, trustworthiness, and accuracy of the answers [12,68]. Nadarzynski et al [12] found that the majority (78%) of the participants were willing to use a chatbot for information and concluded that written information can be better understood than information heard. In addition, an HB could have the advantage that information could be recalled at home at any time after the consultation, in case the specificities were forgotten owing to nervousness or forgetfulness [12]. A few concerns such as the replacement of dietitians, reliability of responses, and lack of responsibility in dealing with the HB were mentioned as well. Some people even indicated having no concerns at all about using the HB. In addition, the items addressing the willingness to share information with and seek information from the HB now and in the future can be interpreted as existing trust in the HB. That the idea of HBs is appreciable and that further development should be pursued were mentioned in the survey. Overall satisfaction with the HB was scored well. Taken together, this reflects the statements shared during the interviews; the strengths of the HB mentioned during the interviews concerned the actual product and idea (eg, the ease of use, practicability, and accessibility), whereas the perceived weaknesses concerned the current development status (eg, design, missing examples, and a lack of the HB’s knowledge), which seems promising for future development steps of the HB. The mentioned topics to be included in the HB are strongly related to everyday life, such as how to specifically plan a meal or which food product is suitable in which situation. This is consistent with the findings of the study by Robinson et al [64], in which specific tips for meals and support in everyday life were identified as benefits that patients with obesity desired from digital tools. Overall, the perception of the HB is positive in terms of trust and strengths, which can be underlined for general eHealth use in the preoperative and postoperative bariatric setting [69].

#### Ratings of the Answers of the HB

It makes sense that the scores are slightly worse when including the category “others” in the calculations. Whenever HB gets asked a question it is not trained on yet, the chance that the answer is not correct or not shown (displayed as “no answer found”) is high. Worse ratings are likely to be given by participants for wrong, inappropriate, or nonexistent answers. The best ratings on “dumping syndrome” and “protein” can be explained by the material that the HB was trained on. It dedicated separate chapters to these 2 topics; therefore, the HB could be trained in detail on them. The category “food tolerance” was rated the worst overall. The material on this topic used for training the HB did not go into details and was more general. Food tolerance and intolerance in general and especially after a bariatric surgery are extremely individual; therefore, if the training material on these topics is general and somewhat unspecific, it can cause the provision of unsatisfying answers to the participants. Boczar et al [70] also discovered some difficulties in generating appropriate answers to FAQs with an AI virtual assistant for assisting individuals undergoing plastic surgery. However, the AI virtual assistant was seen to be able to understand the FAQs of patients undergoing plastic surgery well, which seems promising for future use in health care [70].

### Limitations and Potential Risks

The second part of the questionnaire, although based on several proven-useful English-language questionnaires, was not tested for the quality criteria with the exact composition that it had in this study. The fact that this was a cross-sectional study is seen as a limitation in the methodology. The author’s presence during the study may have caused some bias owing to participants wanting to portray themselves well, and limited openness or honesty may lead to less critical responses [71]. In addition, the sample of 12 participants is relatively small for the statistical analyses of the questionnaires. However, for a usability test, the sample size is sufficient for the first cycle of the iterative process [72,73].

The use of an HB might be a promising approach to address nutrition-related questions in everyday clinical practice. However, there are also potential risks, which must be considered. When patients use an AI-based digital information tool without surveillance by a health care professional, there is a certain risk of misunderstanding or misinterpretation the provided answer [74]. Furthermore, the HB for patients with obesity only covers bariatric nutrition–related questions. Thus, any other comorbidities that require nutritional adaptations are not considered, and patients need to be made aware of this. Another potential risk is digital exclusion. People with low literacy, cognitive impairment, or no access to digital tools should not be at a disadvantage [20,75]. Therefore, the HB must be easy and intuitive to use, and high-quality traditional health care must remain accessible [20].

### Future Work

The HB has a great potential for further development. The next steps include the improvement of the accuracy of the answers, expansion of the topics, and improvement of the presentation of the answers and the design. Subsequently, a further review with a similarly large sample of potential users is needed. A randomized controlled trial with a larger sample would be needed to analyze potential benefits, such as better patient care or improved nutrition knowledge in patients in practice. Beyond exploring the short-term use of an HB around bariatric surgery, exploring more extensive use after surgery would be needed. Nutrition questions might change over the years, and an HB that supports patients in this trajectory could be a preventive tool for weight gain after surgery [76].

Existing interventions using conversational agents focus more on healthy lifestyle behaviors and less on health care setting with patients [77,78]. A recent review showed that chatbot interventions are supportive for physical activity behavior, fruit and vegetable consumption, sleep duration, and sleep quality [77]. Therefore, chatbots also offer the potential to support health care delivery in an efficient, appealing, and personalized manner. This should be explored in areas where lifestyle or behavioral changes are prescribed as part of the treatment, such as rehabilitation and dietetics, and to promote patient compliance. In the future, capturing health professionals’ perceptions of the HB and their willingness to use it in the medical setting would be important. To implement new technologies in patient care, health professionals’ opinions are just as relevant as patients’ opinions. The last hurdle for the use of HBs in practice would their financing and certification as medical devices.

### Conclusions

In this study, the strengths of an HB supporting nutritional care for patients with obesity, such as its satisfactory usability and provision of nutrition information, were determined. Weaknesses were identified in the accuracy of the response of, limited knowledge of, and design of the HB.

## Appendix

Multimedia Appendix 1 Example of a Health Bot generated answer.

Multimedia Appendix 2 Spearman correlation and its *P* value for each category and digital affinity.

## Abbreviations

AI: artificial intelligence
FAQ: frequently asked question
HB: health bot
SUS: System Usability Scale
