# Supplementary figures and images for: Usability and Overall Perception of a Health Bot for Nutrition-Related Questions for Patients Receiving Bariatric Care: Mixed Methods Study
Source: JMIR Hum Factors. 2023 Nov 8;10:e47913. doi: 10.2196/47913 (PMC10666014; doi:10.2196/47913)

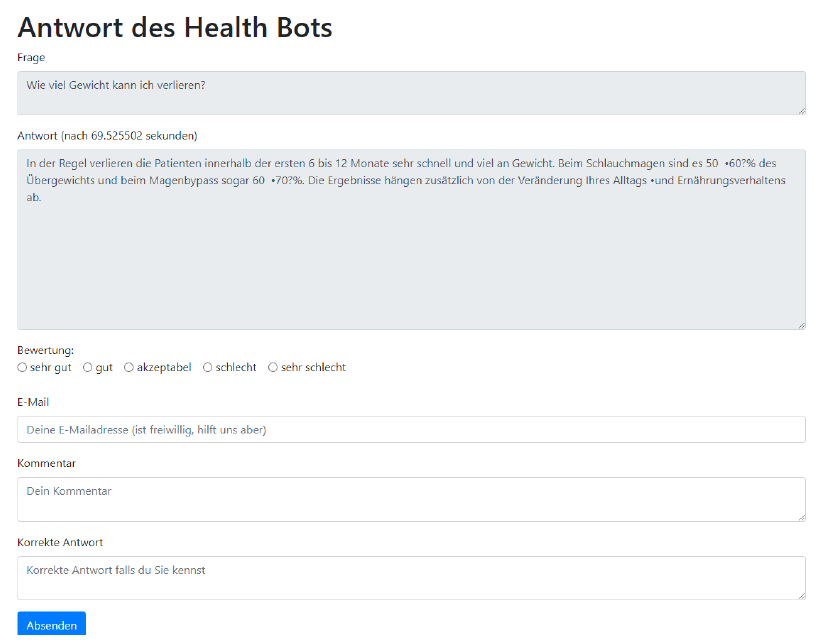

Supplement: Multimedia Appendix 1 [file humanfactors_v10i1e47913_app1.png]
