# Supplementary material for: Usability and Overall Perception of a Health Bot for Nutrition-Related Questions for Patients Receiving Bariatric Care: Mixed Methods Study
Source: JMIR Hum Factors. 2023 Nov 8;10:e47913. doi: 10.2196/47913 (PMC10666014; doi:10.2196/47913)
Supplement: Multimedia Appendix 2 [file humanfactors_v10i1e47913_app2.docx]

Table 6: Spearman correlation and its p-value of each category and digital affinity.

| Category | Spearman correlation | *P*-value of Spearman correlation |
| --- | --- | --- |
| SUS (Usability) | - .19 | .57 |
| Usability | - .09 | .77 |
| Usefulness | - .06 | .85 |
| User-friendliness and Learnability | - .1 | .76 |
| Interface quality | - .26 | .41 |
| Reliability | .25 | .63 |
| Satisfaction | - .20 | .53 |
| Risks | .08 | .82 |
| Benefits | - .08 | .79 |
| Intention to share information | - .18 | .57 |
| Intention to seek information | - .06 | .86 |
